# Item difficulty index, discrimination index, and reliability of the 26 health professions licensing examinations in 2023, Korea: a psychometric study

**DOI:** 10.3352/jeehp.2024.21.40

**Published:** 2024-12-11

**Authors:** Yoon Hee Kim, Bo Hyun Kim, Joonki Kim, Bokyoung Jung, Sangyoung Bae

**Affiliations:** Research and Development Division, Korea Health Personnel Licensing Examination Institute, Seoul, Korea; The Catholic University of Korea, Korea

The Korea Health Personnel Licensing Examination Institute conducts annual national licensing examinations to assess whether candidates possess the minimum competencies required for healthcare practice. Post-examination analyses of item difficulty, discrimination, and reliability are necessary to ensure the validity and appropriateness of these tests. Items with excessively high or low difficulty or discrimination indices may need review and revision, offering valuable feedback to improve future examinations.

This study analyzed the item difficulty, discrimination, and reliability of the 26 health personnel licensing examinations in Korea conducted in 2023 to evaluate their appropriateness. Additionally, it examined the correlation between the average item difficulty index and the average item discrimination index. It is a follow-up report from the 2022 licensing examinations [1].

This was not a human population study, but an analysis of test results. Therefore, institutional review board approval and informed consent were not required.

Item analysis was conducted for the 26 licensing examinations using classical test theory. Item difficulty (P) was calculated as the proportion of examinees selecting the correct answer, ranging from 0 to 1. Item discrimination was assessed through the upper and lower 27% rule, measuring the difference in difficulty between the top 27% and bottom 27% of examinees and the correlation coefficient between the item score and the total test score. The discrimination indices and reliability were calculated only for tests with more than 100 examinees. Statistical analyses were performed using IBM SPSS ver. 21.0 (IBM Corp.).

Pass rates for the 26 examinations varied widely, from 55.2% to 100%. The number of examinees ranged from 6 (health educators–level 1) to 339,417 (care workers). Examinations for physicians, dentists, midwives, nurses, doctors of Korean medicine, and pharmacists had pass rates above 90%. Of note, the midwives’ examination had a 100% pass rate, although only 8 candidates participated (Table 1).

The results of the item analysis for the 2023 examinations are summarized in Table 2.

All examinations demonstrated high reliability, with the lowest reliability observed (0.855) for the health educator (level 3) examination. The average difficulty indices ranged between 62.2% (health educators–level 2) and 81.3% (nurses), while the average item-total correlation spanned from 0.17 (doctors of Korean medicine) to 0.40 (medical technologists and opticians). A negative correlation was observed between the average difficulty index and the average discrimination index (r=–0.3910, P=0.0395), indicating that easier items were less effective at discriminating between high and low performers.

According to classical test theory, average difficulty indices are categorized as moderate (50%–60%), somewhat easy (60%–70%), easy (70%–80%), and very easy (80% or higher). Based on these criteria, the difficulty indices of the 2023 examinations were classified as very easy (nurses and physicians), easy (18 exams), and somewhat easy (10 exams). For medical professionals such as physicians (average difficulty index 80.9%), dentists (77.0%), nurses (81.3%), and doctors of Korean medicine (77.9%), the difficulty indices were in the very easy or easy categories, with pass rates exceeding 90%.

Discrimination indices were interpreted as low (<0.25), average (0.25–0.30), and good (>0.30). Eight examinations showed low discrimination, 9 had average discrimination, and 11 showed good discrimination. For example, the doctors of Korean medicine examination had a low discrimination index (average item-total correlation of 0.17), whereas the optician’s examination showed good discrimination (average item-total correlation of 0.40). All tests demonstrated high reliability, with Cronbach’s α values of 0.855 or higher, ensuring consistent assessment across the examinations.

In 2022, the national licensing examinations had 468,352 examinees with an 89.4% pass rate. In 2023, there were decreases in both examinees (446,054) and passers (388,067), yielding an 87.0% pass rate. The pass rate range shifted from 29.7%–97.1% in 2022 to 52.2%–100% in 2023. An analysis of difficulty indices for both years revealed that most examinations were relatively easy. Thirteen examinations in 2023 exhibited decreased difficulty indices compared to 2022, primarily transitioning among the “easy,” “very easy,” and “somewhat easy” categories, without significant overall shifts. Additionally, the Care Workers Qualification Examination’s transition to computer-based testing in 2023 resulted in non-disclosure of test items and analysis results, limiting direct comparisons.

For discrimination indices, 23 examinations showed lower indices in 2023, and 17 exhibited decreased item-total correlation coefficients. Despite these declines, the overall discrimination indices for the 2023 examinations did not significantly differ from 2022, suggesting no substantial reduction in discriminative power. Confidentiality policies limit the disclosure of detailed item analyses and test results, hindering comparisons with previous analyses and restricting academic discussions on these examinations.

In conclusion, Korea’s 2023 national health personnel licensing examinations demonstrated acceptable ranges of difficulty, discrimination, and reliability indices. However, 8 out of 28 examinations showed low discrimination indices despite appropriate difficulty levels.

## Figures and Tables

**Table 1.** Pass rates for examinees in the 26 national health professions licensing examinations in Korea, 2023

Job titles	No. of examinees	No. of successful candidates	Pass rate (%)
Assistive technology professionals (5th)	330	278	84.2
Care workers^a)^	339,417	299,556	88.3
Dental hygienists (51st)	5,243	4,613	88.0
Dental technicians (51st)	950	787	82.8
Dentists (76th)	794	753	94.8
Dietitians (47th)	5,559	4,032	72.5
Emergency medical technicians–level 1 (29th)	1,594	1,340	84.1
Emergency medical technicians–level 2 (29th)	1,100	896	81.5
Health educators–level 1 (14th)	6	4	66.7
Health educators–level 2 (14th)	131	78	59.5
Health educators–level 3 (14th)	1,009	716	71.0
Health information managers (40th)	2,409	1,588	65.9
Oriental medicine pharmacists (23rd)	154	134	87.0
Medical technologist (51st)	2,958	2,544	86.0
Midwives (34th)	8	8	100.0
Nurses (63rd)	24,015	23,359	97.3
Nursing aides (1st half)	16,156	13,583	84.1
Nursing aides (2nd half)	15,305	12,112	79.1
Occupational therapists (51st)	2,162	1,939	89.7
Opticians (36th)	1,451	977	67.3
Oriental medicine doctors (Doctor of Korean Medicine) (78th)	823	811	98.5
Oriental medicine dispenser (30th)	-	-	
Pharmacists (74th)	2,014	1,887	93.7
Physical therapists (51st)	5,138	4,209	81.9
Physicians (87th)	3,358	3,181	94.7
Prosthetists and orthotists (24th)	115	90	78.3
Radiological technologists (51st)	2,738	2,118	77.4
Rehabilitation counselors–level 1 (7th)	532	462	86.8
Rehabilitation counselors–level 2 (7th)	140	122	87.1
Sanitary technician (45th)	7,685	4,013	52.2
Speech-language pathologists–level 1 (12th)	1,115	710	63.7
Speech-language pathologists–level 2 (12th)	1,645	1,167	70.9

a) In 2023, the Care Workers Qualification Examination transitioned to a computer-based test with continuous availability. For that year only, paper-based testing was conducted concurrently.

**Table 2.** Difficulty index, upper and lower group discrimination index, item-total correlation, and reliability index (Cronbach α) of the 26 health professionals licensing examinations in Korea, 2023

Job title	No. of subjects	No. of items	Difficulty index (%)	Upper and lower group discrimination^a)^	Item-total correlation^a)^	Reliability (Cronbach α)
Assistive technology professional(5th)	2	170	68.2±24.7	0.20±0.14	0.21±0.11	0.876
Care workers^b)^	2	80				
Dental hygienists (51st)	2	200	76.5±15.8	0.26±0.13	0.29±0.12	0.947
Dental technicians (51st)	3	205	74.5±17.5	0.31±0.13	0.34±0.10	0.963
Dentists (75th)	13	364	77.0±22.1	0.18±0.13	0.20±0.10	0.929
Dietitians (47th)	4	220	67.8±20.0	0.33±0.16	0.30±0.13	0.957
Emergency medical technicians–level 1 (29th)	5	230	70.2±18.9	0.27±0.13	0.26±0.10	0.946
Emergency medical technicians–level 2 (29th)	5	140	68.8±21.4	0.24±0.13	0.24±0.09	0.901
Health educators–level 1 (14th)	3	60	64.2±29.5	-	-	-
Health educators–level 2 (14th)	8	180	62.2±23.0	0.27±0.19	0.25±0.15	0.921
Health educators–level 3 (14th)	4	110	66.5±19.9	0.28±0.14	0.27±0.11	0.855
Health information managers (40th)	3	230	67.6±19.2	0.36±0.17	0.34±0.12	0.968
Medical technologists (51st)	4	280	77.8±16.5	0.34±0.15	0.40±.12	0.980
Midwives (34th)	4	165	74.2±23.8	-	-	-
Nurses (63rd)	8	295	81.3±19.5	0.18±0.13	0.24±0.10	0.940
Nursing aides (1st half)	4	100	78.2±17.1	0.34±0.19	0.38±0.13	0.941
Nursing aides (2nd half)	4	100	73.2±20.8	0.33±0.17	0.36±0.13	0.930
Occupational therapists (51st)	4	240	76.8±18.7	0.26±0.15	0.28±0.11	0.954
Opticians (36th)	4	250	69.0±17.3	0.42±0.15	0.40±0.12	0.979
Oriental medicine doctors (Doctor of Korean Medicine) (78th)	11	340	77.9±21.0	0.15±0.11	0.17±0.10	0.911
Oriental medicine pharmacist (24th)	3	250	72.5±21.1	0.27±0.14	0.30±0.15	0.958
Pharmacists (74th)	4	350	73.5±21.6	0.20±0.11	0.25±0.11	0.954
Physical therapists (51st)	5	260	72.0±19.6	0.29±0.13	0.31±0.12	0.963
Physicians (87th)	3	320K	80.9±19.7	0.17±0.13	0.23±0.10	0.941
Prosthetists and orthotists (24th)	7	210	70.3±18.2	0.31±0.18	0.29±0.14	0.952
Radiological technologists (51st)	4	250	71.7±18.7	0.35±0.15	0.33±0.11	0.972
Rehabilitation counselors–level 1 (7th)	7	150 ^c)^	72.5±20.5	0.24±0.16	0.27±0.11	0.916
Rehabilitation counselors–level 2 (7th)	7	120 ^d)^	74.4±20.4	0.28±0.15	0.33±0.12	0.919
Sanitary technicians (45th)	6	220	62.3±21.4	0.33±0.16	0.30±0.13	0.957
Speech-language pathologists–level 1 (12th)	6	140	64.9±21.3	0.28±0.16	0.24±0.12	0.907
Speech-language pathologists–level 2 (12th)	5	150	72.5±20.5	0.36±0.14	0.33±0.12	0.950

Values are presented as mean±standard deviation unless otherwise stated.

a) Discrimination indices and reliability were not calculated for examinations with fewer than 100 candidates.

b) The item analysis results for the Care Workers Qualification Examination are not disclosed.

c) In 2023, the number of test items for rehabilitation counselors (level 1, 7th) was adjusted from 120 to 150.

d) In 2023, the number of test items for rehabilitation counselors (level 2, 7th) was adjusted from 150 to 120.
